# Supplementary figures and images for: Metabolite exchange between microbiome members produces compounds that influence Drosophila behavior
Source: eLife. 2017 Jan 9;6:e18855. doi: 10.7554/eLife.18855 (PMC5222558; doi:10.7554/eLife.18855)

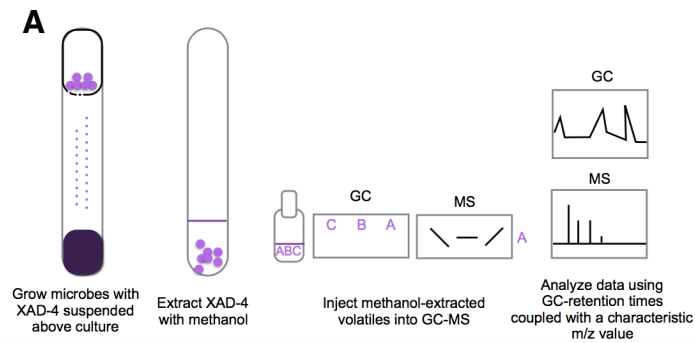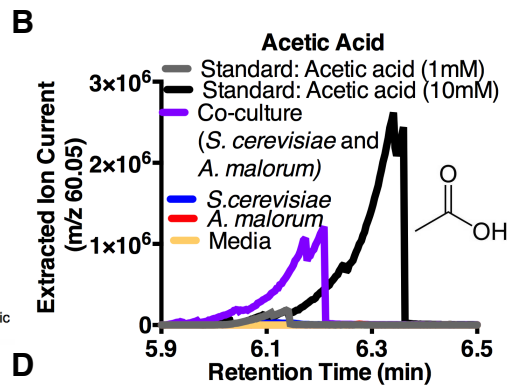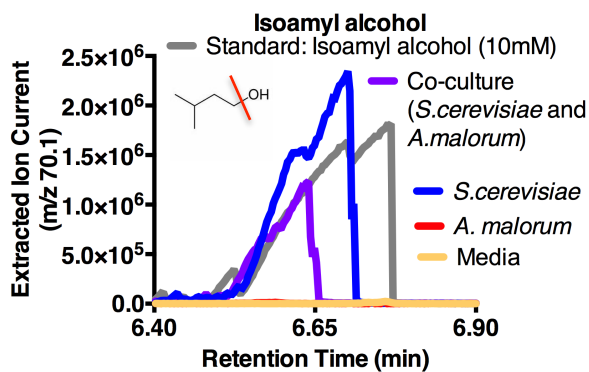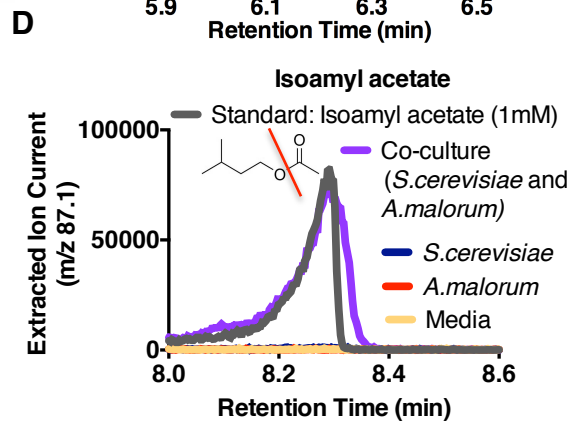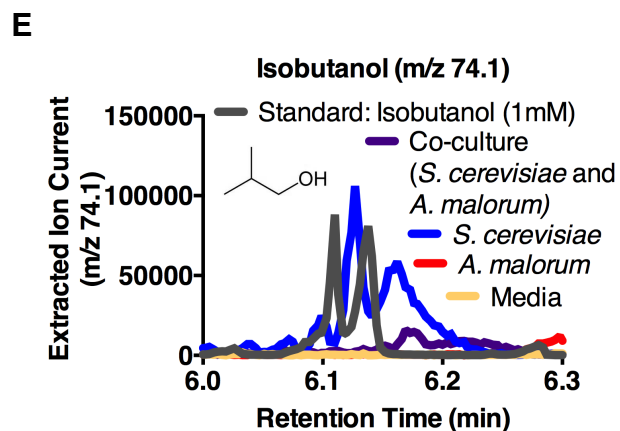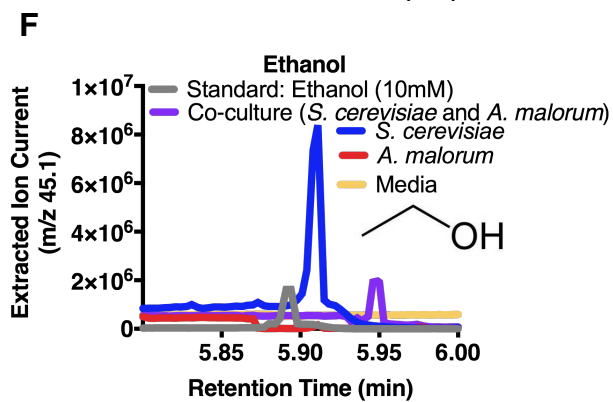

Supplement: Table 1—source data 1. — Extracted ion chromatograms of the five metabolites detected by gas chromatography- mass spectrometry (GC-MS). (A) Schematic depicting the experimental setup (B-F) Representative extracted ion chromatograms from one replicate (out of three total) of one experiment (out of 3–4 total) of m/z values corresponding to major metabolites identified in the experimental conditions along with appropriate standards. Acetic acid (B), isoamyl alcohol (C), isoamyl acetate (D) isobutanol (E), and ethanol (F) were identified as the five major metabolites in the co-culture (S. cerevisiae and A. malorum). Isoamyl alcohol (C), ethanol (E), and isobutanol (F) were identified as the major metabolites in S. cerevisiae grown alone. Extracted ion chromatograms were constructed using the m/z value in the title of each graph. For acetic acid and isobutanol, the m/z value used corresponds to the molecular weight of the molecule. For ethanol, the m/z used corresponds to the molecular weight minus one (hydrogen). For isoamyl alcohol, the m/z used corresponds to the loss of the hydroxyl group (depicted), which may have picked up hydrogen and been lost as water. For isoamyl acetate, the m/z value corresponds to the molecule shown within the graph. In all cases, figures showing the complete mass spectra between the metabolite and standard are found in Table 1—source data 2. Microorganisms were grown 72–96 hr. DOI: http://dx.doi.org/10.7554/eLife.18855.019 [file elife-18855-table1-data1.pdf]

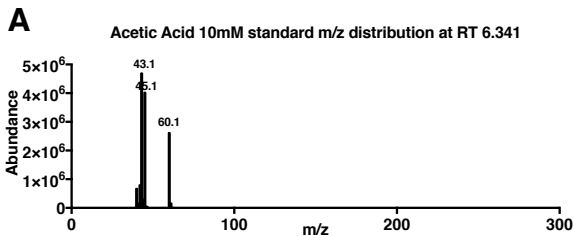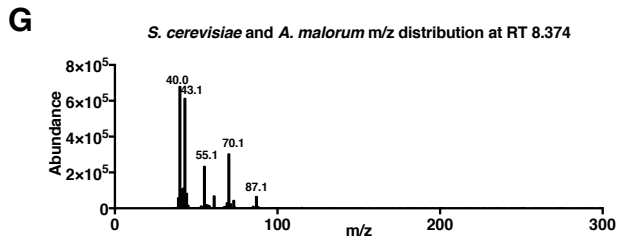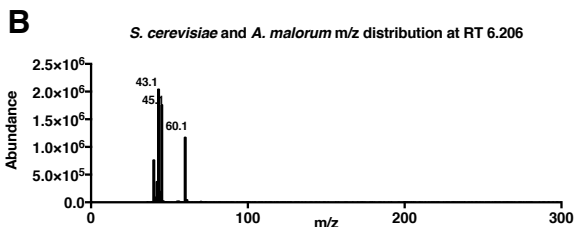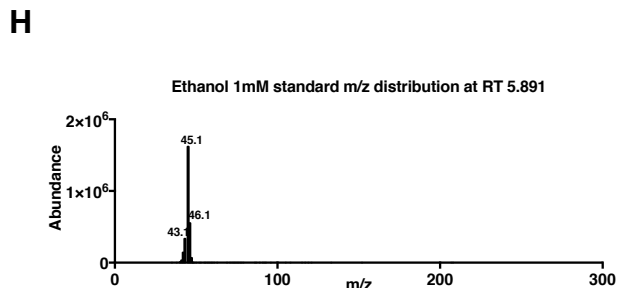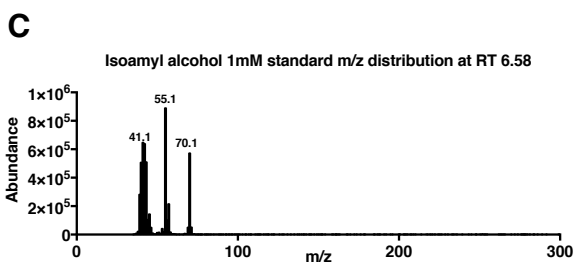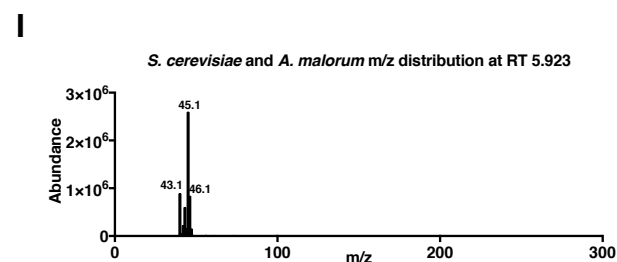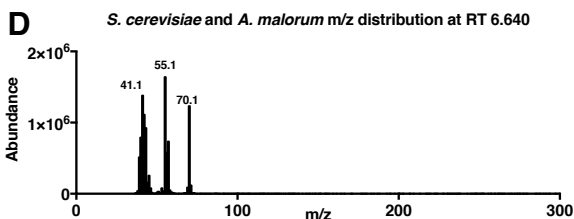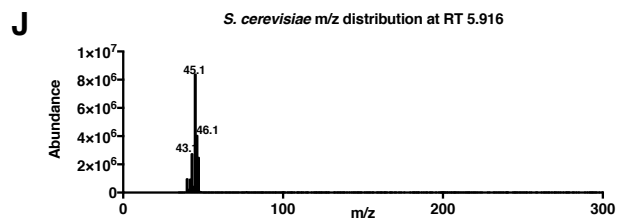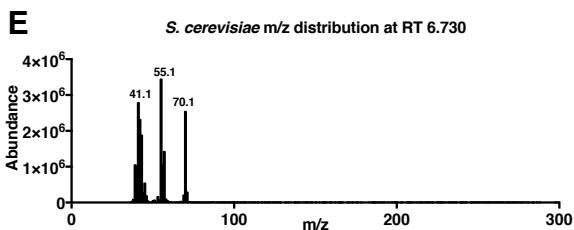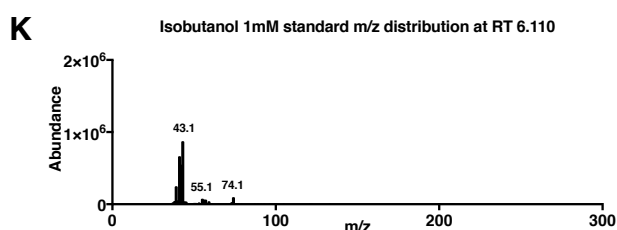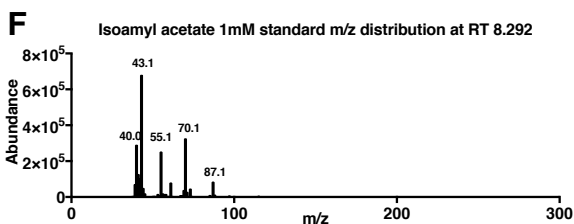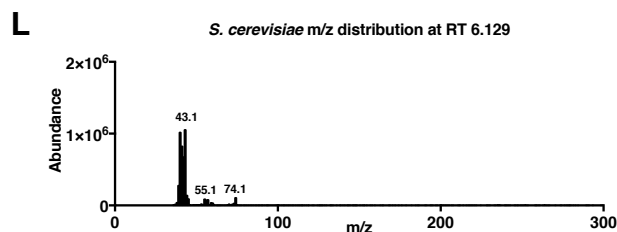

Supplement: Table 1—source data 2. — Representative spectra of acetic acid (A-B), isoamyl alcohol (C-E), isoamyl acetate (F-G), ethanol (H-J) and isobutanol (K-L) in standard and experimental samples. Standard concentrations are denoted on individual graphs. All mass spectra are one replicate (out of 3–4 experiments with three replicates per experiment). DOI: http://dx.doi.org/10.7554/eLife.18855.020 [file elife-18855-table1-data2.pdf]

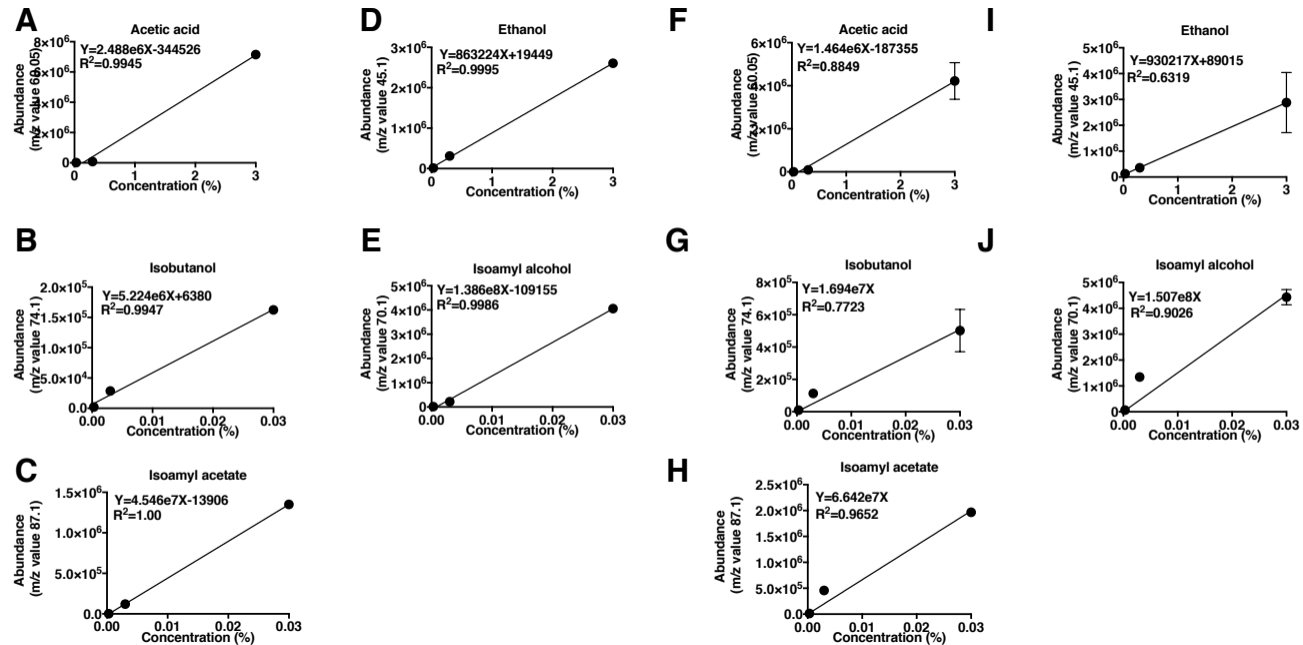

Supplement: Table 1—source data 3. — Estimation of volatile quantity using GC-MS. Separate experiments are graphed in panels (A-E) and (F-J). (A-E) Data points represent the value of a single replicate per concentration for each standard. The abundance of a single m/z value at a specific retention time was chosen for each standard. The values were fitted with a linear regression and the equation was used to estimate the concentration of the five metabolites in the experimental samples from the same experiment. (F-J) Data points represent the mean ± SEM of three replicates for a given concentration for each standard. The abundance of a single m/z value at a specific retention time was chosen for each standard. The values were fitted with a linear regression. The equation was used to estimate the concentration of the five metabolites in the experimental samples from the same experiment. When applicable an equation was calculated when the line was forced to go through X,Y = 0,0; these equations were used to calculate the concentrations of isoamyl alcohol, isoamyl acetate, and isobutanol. DOI: http://dx.doi.org/10.7554/eLife.18855.021 [file elife-18855-table1-data3.pdf]

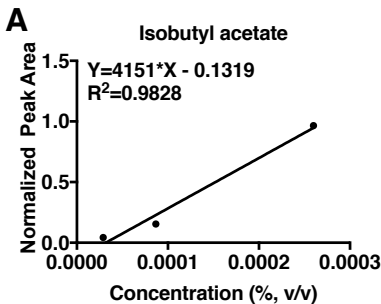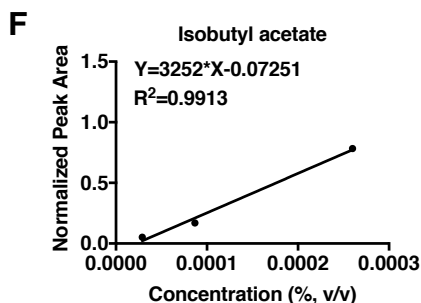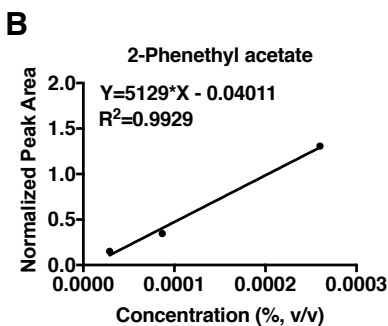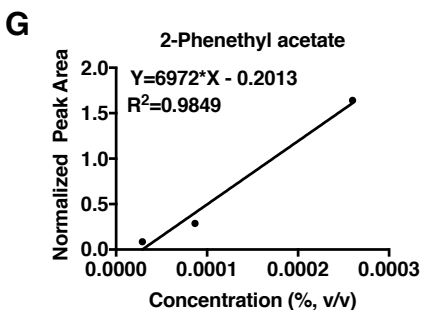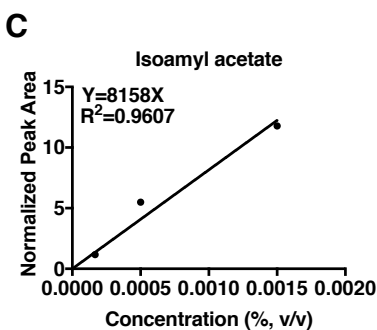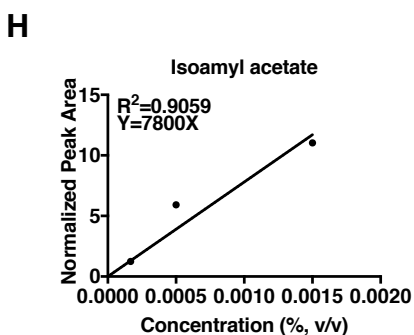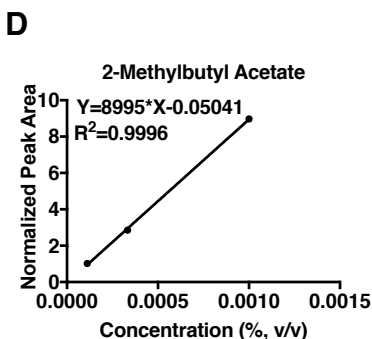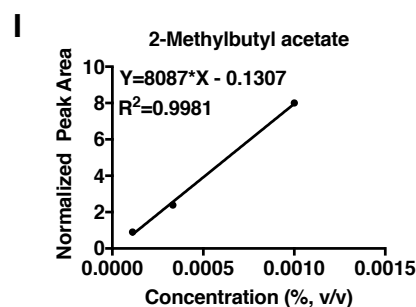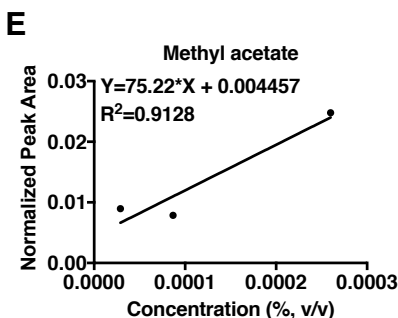

Supplement: Table 2—source data 2. — Normalized peak areas corresponding to metabolites in a defined metabolite mixture (from SPME GC-MS). A linear regression was calculated to quantify the metabolites in the co-culture. Each concentration is from one replicate. A-E and F-I are two separate experiments. Linear regression was used to estimate the concentration of the metabolites in the co-culture containing S. cerevisiae and A. malorum (Table 2) and to complement the co-culture containing A. pomorum adhA (Figure 4C). DOI: http://dx.doi.org/10.7554/eLife.18855.031 [file elife-18855-table2-data2.pdf]
