# Supplementary material for: Metabolite exchange between microbiome members produces compounds that influence Drosophila behavior
Source: eLife. 2017 Jan 9;6:e18855. doi: 10.7554/eLife.18855 (PMC5222558; doi:10.7554/eLife.18855)
Supplement: Table 2—source data 1. — Extracted ion chromatograms of differentially emitted or unique metabolites in the co-culture according to solid phase microextraction gas chromatography-mass spectrometry (SPME GC-MS). Specific metabolites are displayed above each panel. For each panel, the left-most plot compares the co-culture containing S. cerevisiae and A. malorum to S. cerevisiae grown alone, A. malorum grown alone, or media (AJM [apple juice medium]); the right-most plot compares the co-culture containing S. cerevisiae and A. pomorum wild-type to the co-culture containing S. cerevisiae and A. pomorum adhA, since A. pomorum adhA is required for Drosophila co-culture preference (Figure 5A). The two plots within the same panel contain the same standard. The y-axis for each plot is the ion current for a m/z value that discriminates the metabolite of interest over a specific retention time window. The following m/z values were chosen for each metabolite based on standards or, in the cases of putative and unknown metabolites (I and J) were chosen from the experimental groups: (A) m/z 74.04 (B) m/z 88.08 (C) m/z 73.03 (D) 87.05 (E) 74.02 (F) 104.04 (G) 60.05 (H) 88.05 (I) 101.06 (J) 101.06. Each panel is one representative replicate of 1 experiment (out of 3–5 total replicates in three experiments). DOI: http://dx.doi.org/10.7554/eLife.18855.030 [file elife-18855-table2-data1.pdf]

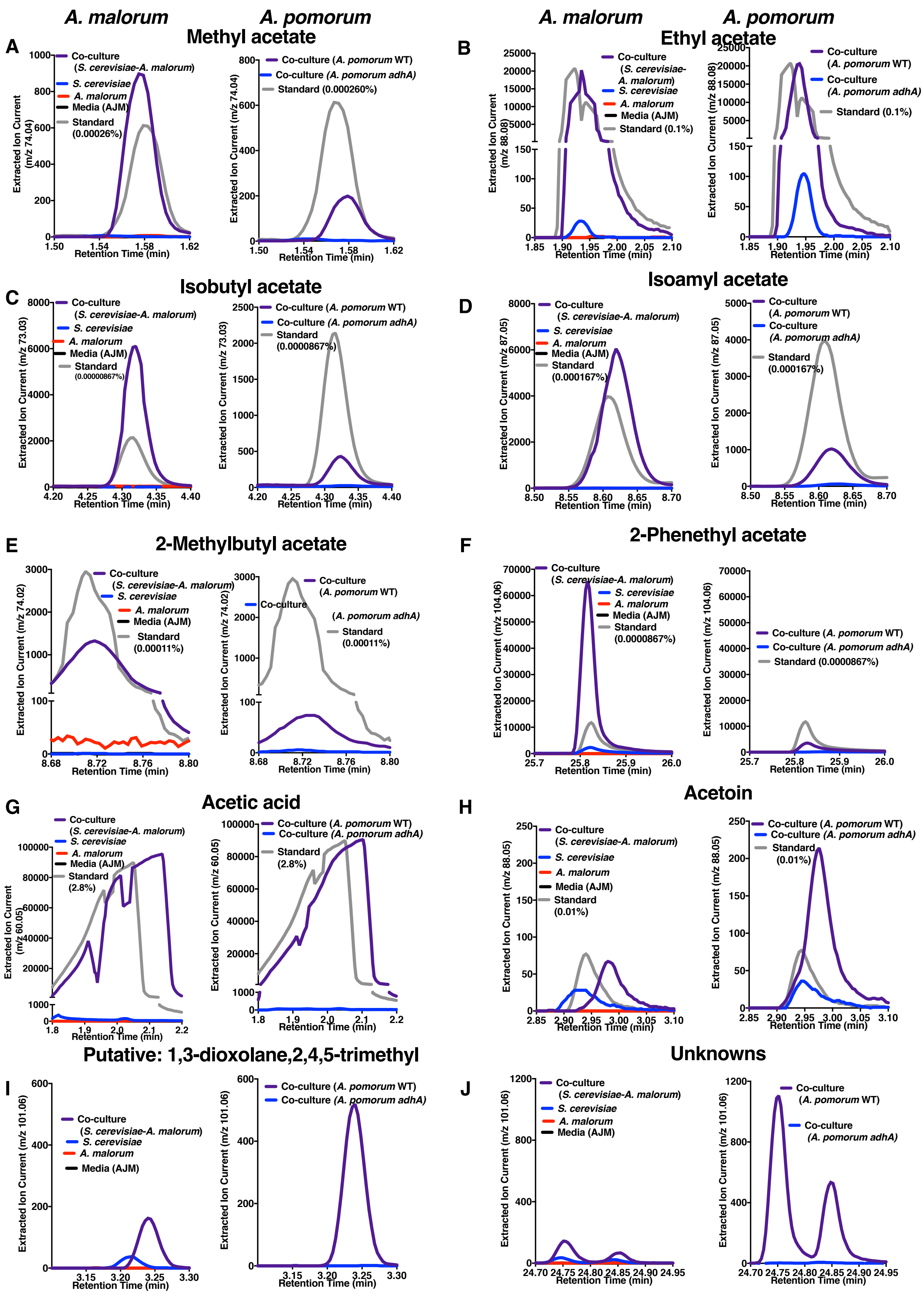

A. malorum
A. pomorum
A. malorum
A. pomorum
A
Methyl acetate
Co-culture
(S. cerevisiae-A. malorum)
S. cerevisiae
A. malorum
Media (AJM)
Standard
(0.00026%)
Co-culture (A. pomorum WT)
Co-culture (A. pomorum adhA)
Standard (0.000260%)
Extracted Ion Current (m/z 74.04)
Retention Time (min)
B
Ethyl acetate
Co-culture
(S. cerevisiae-
A. malorum)
S. cerevisiae
A. malorum
Media (AJM)
Standard (0.1%)
Co-culture
(A. pomorum WT)
Co-culture
(A. pomorum adhA)
Standard (0.1%)
Extracted Ion Current (m/z 88.08)
Retention Time (min)
C
Isobutyl acetate
Co-culture
(S. cerevisiae-A. malorum)
S. cerevisiae
A. malorum
Media (AJM)
Standard
(0.00000867%)
Co-culture (A. pomorum WT)
Co-culture (A. pomorum adhA)
Standard
(0.0000867%)
Extracted Ion Current (m/z 73.03)
Retention Time (min)
D
Isoamyl acetate
Co-culture
(S. cerevisiae-A. malorum)
S. cerevisiae
A. malorum
Media (AJM)
Standard
(0.000167%)
Co-culture
(A. pomorum WT)
Co-culture
(A. pomorum adhA)
Standard
(0.000167%)
Extracted Ion Current (m/z 87.05)
Retention Time (min)
E
2-Methylbutyl acetate
Co-culture
(S. cerevisiae-A. malorum)
S. cerevisiae
A. malorum
Media (AJM)
Standard
(0.00011%)
Co-culture
(A. pomorum WT)
Co-culture
(A. pomorum adhA)
Standard
(0.00011%)
Extracted Ion Current (m/z 74.02)
Retention Time (min)
F
2-Phenethyl acetate
Co-culture
(S. cerevisiae-A. malorum)
S. cerevisiae
A. malorum
Media (AJM)
Standard
(0.0000867%)
Co-culture (A. pomorum WT)
Co-culture (A. pomorum adhA)
Standard (0.0000867%)
Extracted Ion Current (m/z 104.06)
Retention Time (min)
G
Acetic acid
Co-culture
(S. cerevisiae-A. malorum)
S. cerevisiae
A. malorum
Media (AJM)
Standard
(2.8%)
Co-culture (A. pomorum WT)
Co-culture (A. pomorum adhA)
Standard
(2.8%)
Extracted Ion Current (m/z 60.05)
Retention Time (min)
H
Acetoin
Co-culture
(S. cerevisiae-A. malorum)
S. cerevisiae
A. malorum
Media (AJM)
Standard
(0.01%)
Co-culture (A. pomorum WT)
Co-culture (A. pomorum adhA)
Standard
(0.01%)
Extracted Ion Current (m/z 88.05)
Retention Time (min)
I
Putative: 1,3-dioxolane,2,4,5-trimethyl
Co-culture
(S. cerevisiae-A. malorum)
S. cerevisiae
A. malorum
Media (AJM)
Co-culture (A. pomorum WT)
Co-culture (A. pomorum adhA)
Extracted Ion Current (m/z 101.06)
Retention Time (min)
J
Unknowns
Co-culture
(S. cerevisiae-A. malorum)
S. cerevisiae
A. malorum
Media (AJM)
Co-culture
(A. pomorum WT)
Co-culture
(A. pomorum adhA)
Extracted Ion Current (m/z 101.06)
Retention Time (min)
