# Supplementary material for: Metabolite exchange between microbiome members produces compounds that influence Drosophila behavior
Source: eLife. 2017 Jan 9;6:e18855. doi: 10.7554/eLife.18855 (PMC5222558; doi:10.7554/eLife.18855)
Supplement: Supplementary file 2. — DOI: http://dx.doi.org/10.7554/eLife.18855.055 [file elife-18855-supp2.docx]

| **Supplemental File 2.** Microorganisms and their sources used in this study | | | | |  |
| --- | --- | --- | --- | --- | --- |
| **Organism** | **Clade** | | **Source** | |  |
| *Saccharomyces cerevisiae* | Yeast | | *Drosophila pinicola,* Phaff culture collection | |  |
| *S. cerevisiae* Y9 | Yeast | | Ragi (similar to Sake wine) fermentation (30) | |  |
| *S. cerevisiae* YB-210 | Yeast | | Banana; ARSC culture collection (68) | |  |
| *Pichia membranefaciens* | Yeast | | *D. melanogaster* in Handelsman Laboratory | |  |
| *Candida californica* | Yeast | | Bananas and *D. melanogaster* habitat in Stamps laboratory; Phaff culture collection | |  |
| *Hanseniaspora uvarum* | Yeast | | Bananas and *D. melanogaster* habitat in Stamps laboratory; Phaff culture collection | |  |
| *Lactobacillus plantarum* cs | LAB | | *D. melanogaster* in Handelsman Laboratory (25) | |  |
| *L. brevis*_lab | LAB | | *D. melanogaster* in Handelsman Laboratory | |  |
| *L. fermentum* | LAB | | *D. melanogaster* in Handelsman Laboratory | |  |
| *L. brevis*_wild | LAB | | Wild-caught *D. melanogaster* (19) | |  |
| *Leuconostoc* sp_Fabian | LAB | | Wild-caught *D. melanogaster* (69) | |  |
| *L. plantarum* | LAB | | *D. melanogaster* in Handelsman Laboratory | |  |
| *Leuconostoc durionis* | LAB | | Wild-caught *D. melanogaster* (69) | |  |
| *Acetobacter pasteurianus*_lab | AAB | | *D. melanogaster* in Handelsman Laboratory | |  |
| *A. cerevisiae* | AAB | | *D. melanogaster* in Handelsman Laboratory | |  |
| *A. malorum* | AAB | | *D. melanogaster* in Handelsman Laboratory | |  |
| *A. indonensiensis* | AAB | | *D. melanogaster* in Handelsman Laboratory | |  |
| *A. pasteurianus*_wild | AAB | | Wild-caught *D. melanogaster* (19) | |  |
| *A. orleanensis* | AAB | | Wild-caught *D. melanogaster* (19) | |  |
| *A. pomorum* | AAB | | *D. melanogaster* in Won-Jae Lee Laboratory (35) | |  |
| *A. pomorum adhA* | | AAB | | Mutant derivative of *A. pomorum* (35) | |

LAB = lactic acid bacteria; AAB = acetic acid bacteria
